# A Method Based on Nanobodies Against AMH to Establish Immunochromatography for Evaluation of Follicular Development in Bactrian Camels

**DOI:** 10.3390/vetsci13080788

**Published:** 2026-08-07

**Authors:** Li Wang, Yaqian Jin, Guoli Zhang, Xue Chen, Hongrui Ren, Lanjie Li, Jingjing Tian, Bayier Mengke, Peiming Li, Yongjia Ma, Caolemeng Qiqige, Wa Gao, Guiqin Liu, Ruiwen Fan

**Affiliations:** 1 College of Agriculture and Biology, Liaocheng University, Liaocheng 252000, China; lzm17391684885@163.com (L.W.); jinyaqian@lcu.edu.cn (Y.J.); 19993447306@163.com (G.Z.); chenxue@lcu.edu.cn (X.C.); lilanjie@lcu.edu.cn (L.L.); 2 College of Veterinary Medicine, Shanxi Agricultural University, Jinzhong 030801, China; ren18740121@163.com (H.R.); tjj2587336883@163.com (J.T.); 3 Animal Husbandry and Veterinary Technology Extension Center of Alxa in Inner Mongolia, Alxa 750300, China; amxmsyzmk@163.com; 4 Technical Department of Ningbo Sansheng Biotechnology Co., Ltd., Ningbo 315171, China; tj1531@126.com; 5 Animal Disease Prevention and Control Center of Alxa Left Banner, Alxa 750300, China; 18648303336@163.com; 6 Comprehensive Service and Support Center for Agriculture and Animal Husbandry in Jilantai Town of Alxa Left Banner, Alxa 750300, China; jltsyz2007@126.com (C.Q.); 18604830344@163.com (W.G.)

**Keywords:** anti-Müllerian hormone (AMH), Bactrian camel, follicle, immunochromatography, nanobody

## Abstract

(1) Background: Anti-Müllerian hormone (AMH) is a key indicator for predicting ovarian reserve and fertility. Currently, B-mode ultrasound examination is the primary method for evaluating follicular development in Bactrian camels, although it has several limitations. This study aims to develop a colloidal gold strip based on AMH nanobody preparation, establishing an efficient, simple, and rapid detection method suitable for evaluating follicular development. (2) Methods: Two nanobodies specifically binding to AMH were screened and identified from an alpaca naive nanobody library using phage display technology. The nanobodies were expressed in the yeast expression vector (pMCO-AOXα) and purified by affinity chromatography on a nickel column. Based on the purified nanobodies, a colloidal gold immunochromatographic strip was constructed for detecting serum AMH levels. (3) Results: In this study, anti-AMH nanobodies (AMH-VHH-A1 and AMH-VHH-A10) were expressed and purified; these were successfully utilized to prepare colloidal gold immunochromatographic strips for detecting AMH in serum. The T-line intensity of the colloidal gold detection was positively correlated with the number of growing follicles obtained by B-mode ultrasound imaging. (4) Conclusions: The colloidal gold strips based on nanobodies against AMH established in this research provided an effective, simple, rapid, and on-site method for evaluating follicular development, thus supplying a reference for reproductive management in Bactrian camels.

## Simple Summary

Anti-Müllerian hormone (AMH) is critical for assessing ovarian reserve, yet rapid on-site detection methods for follicular development in Bactrian camels remain unavailable. In the present work, we developed colloidal gold immunochromatographic strips using two novel nanobodies (AMH-VHH-A10 and AMH-VHH-A1) screened from an alpaca naive library via phage display. The strips could effectively detect serum AMH levels which positively correlated with growing follicle counts under B-mode ultrasound, thus supplying a simple, rapid, and on-site-applicable tool for reproductive management in Bactrian camels. Future studies will expand sample sets to improve detection, optimize test strip performance, and facilitate implementation of this technology within breeding evaluation systems.

## 1. Introduction

Anti-Müllerian hormone (AMH), also known as Müllerian inhibiting substance (MIS), is a glycoprotein belonging to the transforming growth factor-beta (TGF-β) subfamily of ligands [1]. The TGF-β family comprises multifunctional polypeptide growth factors, suggesting that AMH plays diverse roles. AMH is naturally produced by granulosa cells of growing follicles [2] and regulates multiple ovarian processes, including inhibition of primordial follicle activation and preantral follicle growth [3]. Human AMH is synthesized as a proprotein (proAMH) consisting of 560 amino acids (aa), and it requires proteolytic processing for activation [4]. The N-terminal region (AMHN, with a molecular weight of 57 kDa) and the C-terminal region (AMHC, with a molecular weight of 12 kDa) are responsible for activity regulation and receptor binding, respectively [5]. Research findings indicate that two forms of AMH circulate in the bloodstream: the inactive precursor proAMH and the receptor-binding variants AMHN and AMHC [6]. Homodimers of AMHN and AMHC form a stable noncovalent complex (AMHN, C) that enhances AMH signaling activity [7]. Commercially available assays measure both forms indiscriminately [8], meaning that most existing studies report total AMH levels ([proAMH] + [AMHN, AMHC]) [6]. The structure of AMH is highly conserved across mammalian species, with the bovine variant of AMH consisting of 575 amino acids and sharing 76% amino acid identity with human AMH [9]. Phylogenetic analysis reveals that camels are closer to primates (humans) in terms of AMH sequence than to ruminants [9].

Transduction of the AMH signal occurs through AMHR2, which activates intracellular SMAD proteins along with additional signaling pathways to promote the expression of target genes [10]. In the ovary, AMH inhibits the initial recruitment of primordial follicles (PMFs), and influences growth that depends on follicle-stimulating hormone (FSH) [10]. In addition, AMH interacts with various hormones in the hypothalamic–pituitary–gonadal axis (HPG axis), directly activating gonadotropin-releasing hormone (GnRH) neurons through anti-Müllerian hormone receptor type 2/activin receptor-like kinase 6 (AMHR2/ALK6) [11]. From the perspective of cross-species physiological functions, AMH is involved in regulating the development and homeostasis maintenance of gonads in all vertebrates, and it can also balance the proliferation and differentiation processes of fish germ cells [12]. Additionally, AMH signaling plays a critical role in modulating luteal cell function through both autocrine mechanisms and post-translational processing [13]. In clinical application, AMH can replace transvaginal ultrasound in assessing the morphology of polycystic ovaries, and it is one of the three major diagnostic reference indicators for polycystic ovary syndrome (PCOS) [14]. Evaluating ovarian follicular reserve is a crucial method for predicting an animal’s fertility, and circulating AMH levels serve as a core indicator reflecting ovarian reserve function [15]. The use of AMH as a fertility marker has been demonstrated in cows, sheep, and mares, and has also been indicated in female dogs with similar body weight, with a noteworthy correlation between AMH concentrations and litter size being reported [15]. Furthermore, circulating AMH levels may serve as potential predictors of reproductive viability in gilts, with AMH levels measured on day 160 appearing to be a significant predictive marker [16].

The highly consistent AMH value in dromedaries is a reliable indicator for predicting superovulatory responses and results in dromedaries [17]. There is a strong correlation between serum AMH levels and numbers of developing follicles, leading to a growing recognition of serum AMH levels as an important marker of ovarian reserve [5]. Using the electrochemiluminescence immunoassay kit Elecsys AMH, blood serum AMH levels in alpacas were shown to range from 4.10 to 22 ng/mL, whereas those in llamas ranged from 1.79 to 10.05 ng/mL—demonstrating interspecies differences in AMH concentrations [18].

Assessing the ovarian follicular reserve holds significant importance for predicting fertility, and the circulating AMH is crucial for supporting the ovarian reserve [19,20]. At present, assessment of follicular development in Bactrian camels relies mainly on B-mode ultrasound monitoring; however, this involves significant limitations because the large body of the Bactrian camel presents a challenge to rapid and professional operation on-site. Therefore, developing a portable and rapid on-site detection technology for serum AMH has high practical value for predicting the status of follicular development in Bactrian camels.

## 2. Materials and Methods

### 2.1. Samples Collected for the Experiments

Ovaries of adult Bactrian camels were collected from the slaughterhouse of Alxa Left Banner. Parts of ovaries were put in formaldehyde for paraffin sections, and parts in liquid nitrogen for protein extraction. The same 3 healthy female Bactrian camels were used for both blood collection and B-mode ultrasound detection; these animals were selected at random from a united family farm in Alxa Left Banner. Informed consent was obtained from owners of privately owned animals.

### 2.2. Synthesis of the Antigen of AMH Multi-Peptide

The antigen of AMH peptide (N’-PRASPEPEEAPPSAGGGGSRGRERSGSARAQRSGGGGSQSDRNPRYGNH-C’) was chemically synthesized with purity > 90% by HPLC.

### 2.3. Screening of Nanobodies Against AMH

The synthesized AMH peptide was used to screen nanobodies against AMH from the nanobody phage library in pCANTAB5 (Chinese patent CN201910058785.0) by four rounds, with coating concentrations of AMH peptide of 20 μg/mL, 10 μg/mL, 8 μg/mL, and 5 μg/mL, respectively. After washing, the obtained phage solution was mixed with the TG1 solution to be infected; this was mixed with KM13 auxiliary phage to be infected, and then subjected to a shaking culture at 35~42 °C and centrifugation at 7500~8500 g. The precipitation was resuspended in liquid medium and then subjected to a second shaking culture at 28~32 °C and a second centrifugation at 2000~2100 g. The second supernatant was mixed with a blocking solution, incubated, and then subjected to indirect ELISA detection; this determined the reactivity of the second supernatant with the AMH protein (see Supplementary Table S1).

### 2.4. Expression and Purification of Nanobodies Against AMH

Plasmids of strains with reactivity to the AMH peptide were extracted and sequenced using universal primers, and the sequence was identified as a correct VHH based on the high homology with the standards frame of VHH sequences in Genbank. The VHH fragment including the plasmid upstream primer (5′-TTTCTATTACTAGGCCCAGCCGGCCCAGGTGCAGCTCGTGGAGTCT-3′) and downstream primer (5′-AAACCGTTGGCCATAATGGCCTGAGGAGACGRTGACSTSGGTC-3′) was ligated with the yeast expression vector (pMCO-AOXα) to obtain the recombinant plasmid, which was transformed into Pichia pastoris GS115 to obtain a nanobody expression strain.

The GS115 expression strain was enriched in 100 mL of YPG culture medium at 30 °C, 180 rpm for 12 h, then centrifuged at 6000× *g* for 10 min. The precipitation was re-suspended with 100 mL of BMMY medium to be induced for expression at 28 °C, 180 rpm for 96 h, supplementing with 1 mL of methanol every 24 h. After fermentation, the supernatant was collected by centrifugation at 6000× *g* for 10 min.

The supernatant was filtered using a 0.22 µm filter which was loaded in the His-tag nickel column of an AKTA machine (Cytiva) and eluted with imidazole elution solutions of different concentration gradients. The eluted proteins were collected and subjected to Western blotting detection using secondary antibodies specific to the His-tag, as below.

### 2.5. Distribution and Expression of AMH and AMHR2 in Ovaries of Bactrian Camels Detected by Immunohistochemistry and Western Blotting

The 6 μm paraffin sections of camel ovarian tissues were cut to detect distribution of AMH and AMHR2 by immunohistochemical methods. Briefly, after dewaxing and dehydrating the tissue samples on the slide, the sections were boiled in sodium citrate solution for 10 min to repair the antigen. After cooling at room temperature, the sections were incubated with 3% hydrogen peroxide for 20 min. Subsequently, the sections were incubated with BSA solution at 37 °C for 1 h. Primary antibodies against AMH (AMH-VHH-A10, prepared in this research, diluted at 1:200) and AMHR2 (from rabbit, diluted at 1:200) were used to incubate the slides overnight at 4 °C. The next day, after returning to room temperature, the sections were rinsed three times with PBS, and then incubated with horseradish peroxidase-labeled anti-HIS-tag mouse monoclonal antibody (targeting AMH-VHH-A1, diluted at 1:200) and goat anti-rabbit antibody (targeting AMHR2, diluted at 1:200) at 37 °C for 1 h. The sections were subsequently stained with DAB for 15 min and then with hematoxylin for 30 s. After dehydration and clearing, the sections were sealed and fixed, and observed under a microscope.

A total size of 1 m^3^ of camel ovarian tissues was taken and ground in a RIPA lysis buffer (Beyotime, Shanghai, China) using a grinder for 1.5 min. Total protein was then extracted by centrifugation at 12,000 rpm for 10 min. After separating the protein by 10% SDS-PAGE electrophoresis, proteins were transferred to a nitrocellulose membrane (NC membrane) for Western blot analysis using primary antibodies against AMH (AMH-VHH-A1) and AMHR2 (both diluted to 1:1000) and their corresponding secondary antibodies (His-tag and goat anti-rabbit, respectively).

### 2.6. Establishment of Colloidal Gold Immunochromatography Based on AMH Nanobodies

Glass fiber membranes were immersed separately in the reaction pad solution and gold pad solution for 15 min to obtain reaction pads and gold label pads, which were then placed in a 37 °C constant temperature drying oven overnight.

The AMH-VHH-A1 and AMH-VHH-A10 nanobodies obtained as described above were used to establish colloidal gold immunochromatography test strips, as shown in the following diagram. Briefly, two 1 mL tubes of colloidal gold, each containing 4 µL of potassium carbonate solution, were thoroughly mixed. To these tubes were added 10 µg AMH-VHH-A10 nanobody and 10 µg chicken IgY antibody, respectively, and thorough mixing was carried out again. After standing at room temperature for 20 min, 100 µL of 10% BSA was added and mixed, then allowed to stand for another 20 min. The mixture was then centrifuged at 12,000× *g* for 10 min, the supernatant was discarded, and all precipitates were resuspended with 50 µL of colloidal gold renaturation solution. The suspension was then sprayed onto a gold pad using a XYZ Platform Dispenser (Shanghai Kinbio Tech. Co., Ltd., Shanghai, China), and the gold label pad was completed by drying at 37 °C overnight. Using a XYZ Platform Dispenser, the mixture of the AMH-VHH-A1 nanobody and PBS was deposited onto the NC membrane as the T line, while the mixture of goat anti-chicken IgY antibody and PBS as the C line, respcetively.

**Figure 1:**
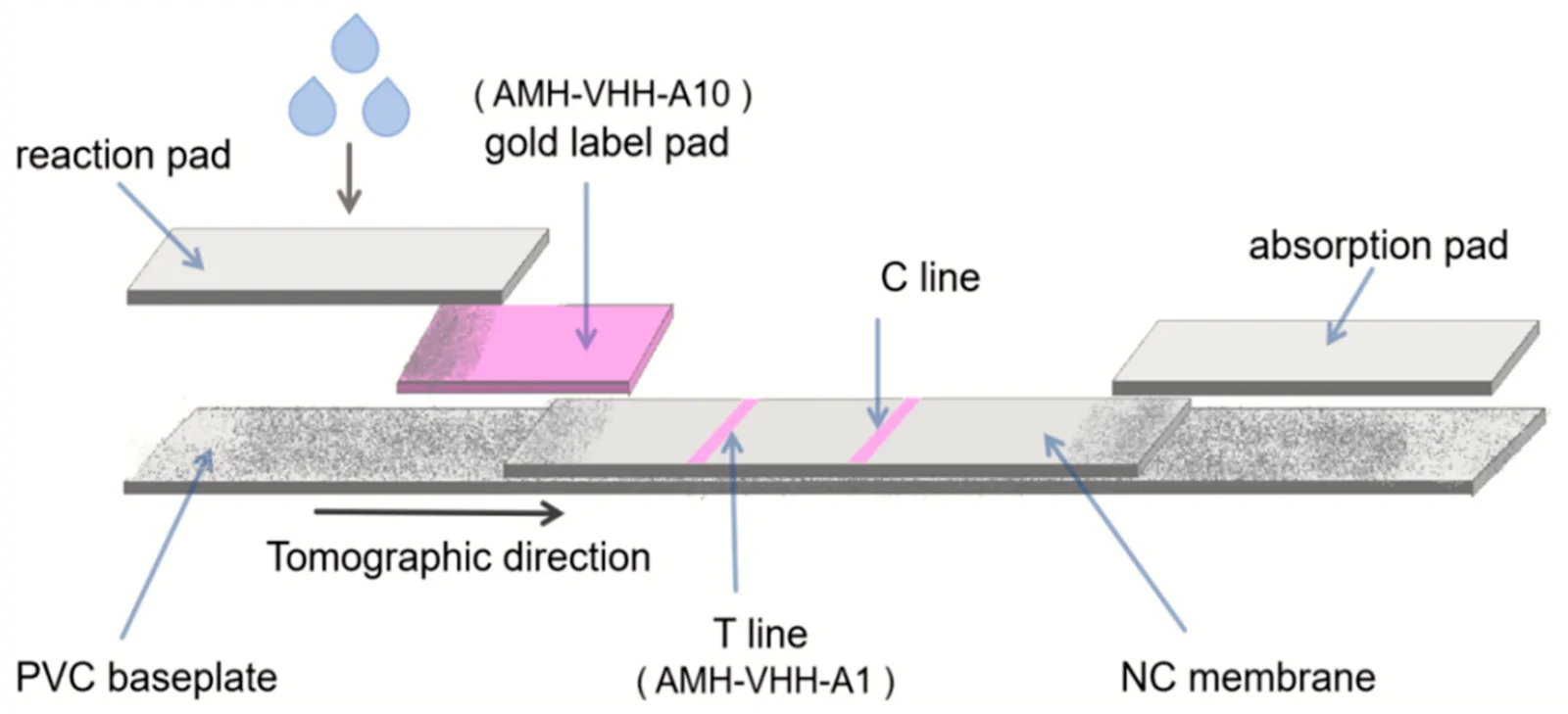
Schematic diagram of colloidal gold immunochromatography strip. On the T line, the mixture of the AMH-VHH-A1 nanobody and PBS was deposited, and on the C line, the mixture of goat anti-chicken IgY antibody and PBS was deposited.

The colloidal gold immunochromatography was assembled on the PVC baseplate in the following order as absorption pad, NC membrane, gold label pad, and reaction pad with a 2 mm overlap between the pads., which was cut into 4 mm wide test strips using a guillotine cutter (Figure 1).

### 2.7. B-Mode Ultrasound Monitoring of Follicular Development in Bactrian Camel Ovaries

The camel was positioned in a standing position, and follicular development in the ovaries was monitored using transrectal B-mode ultrasound. The growing follicles appeared anechoic, allowing observation of their number, diameter, and location.

## 3. Results

### 3.1. Structures of VHH Sequences and Purified VHH Against AMH

**Figure 2:**
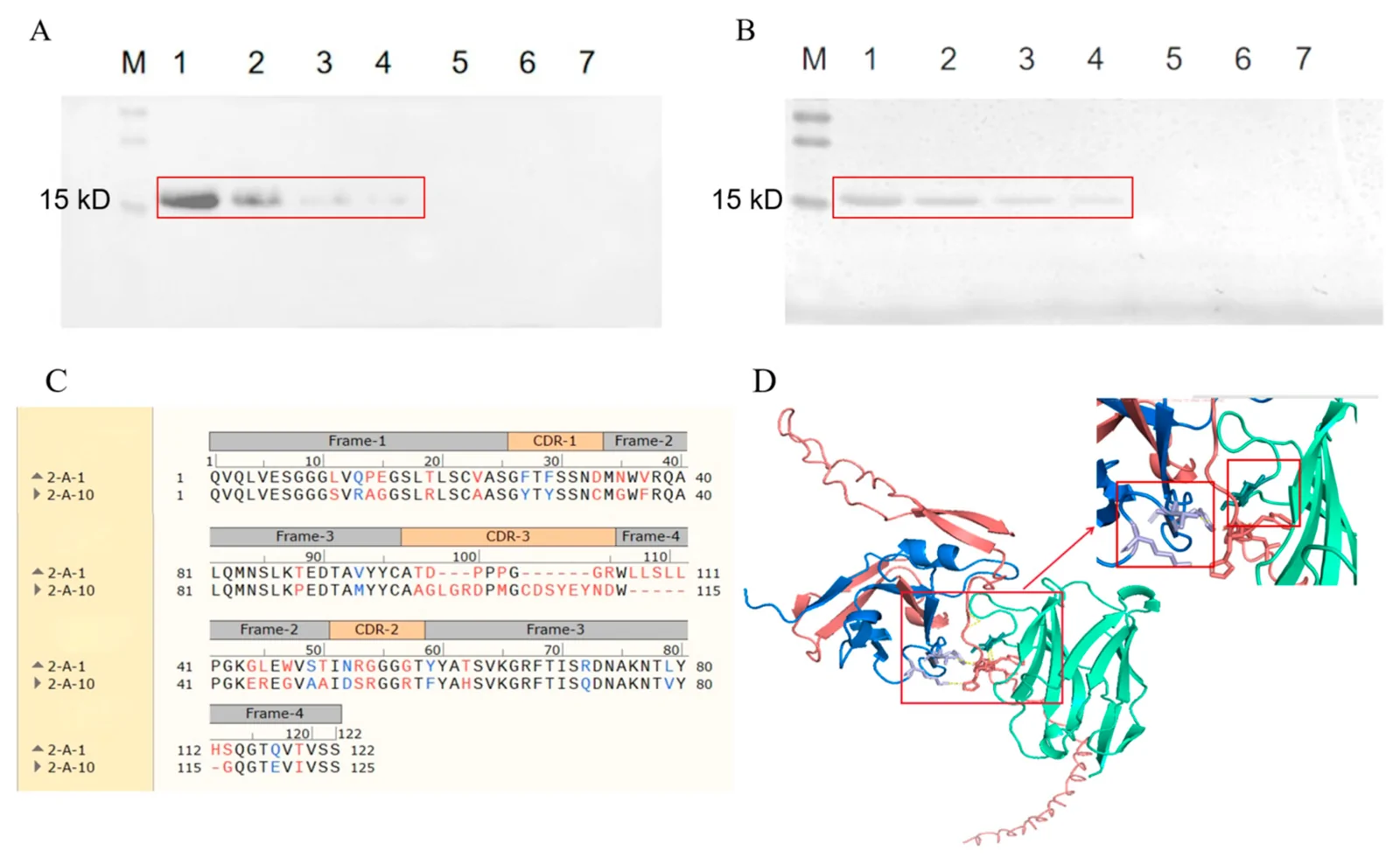
Detection of purified VHHs against AMH by Western blotting. (**A**,**B**) Nanobodies named as VHH-AMH-A1 and VHH-AMH-A10, respectively, were detected by his-tag (lane M: marker; lanes 1~4: 20% eluent; lane 5: 30% eluent; lane 6: 40% eluent; lane 7: 100% eluent). (**C**) Alignments of amino acid sequences of frame regions and CDRs of VHH-AMH-A1 and VHH-AMH-A10. (**D**) Structural simulation and molecular docking: AMH-VHH-A1 (blue), AMH-VHH-A10 (green), and camel AMH (red).

The sequences of VHH against AMH were aligned, showing the frame and the CDR regions. The molecular weights of the purified VHHs against AMH were about 15 kDa with His-tag (Figure 2A,B). The CDR3 regions of both VHHs against AMH were composed of more than seven amino acids (Figure 2C). Using the AlphaFold2 protein structure prediction platform to simulate molecular docking experiments, two specific nanobodies formed noncompetitive pairs with the antigens, with each molecule possessing an independent epitope (Figure 2D).

### 3.2. Distribution and Expression of AMH and AMHR2 in Bactrian Camel Ovaries

**Figure 3:**
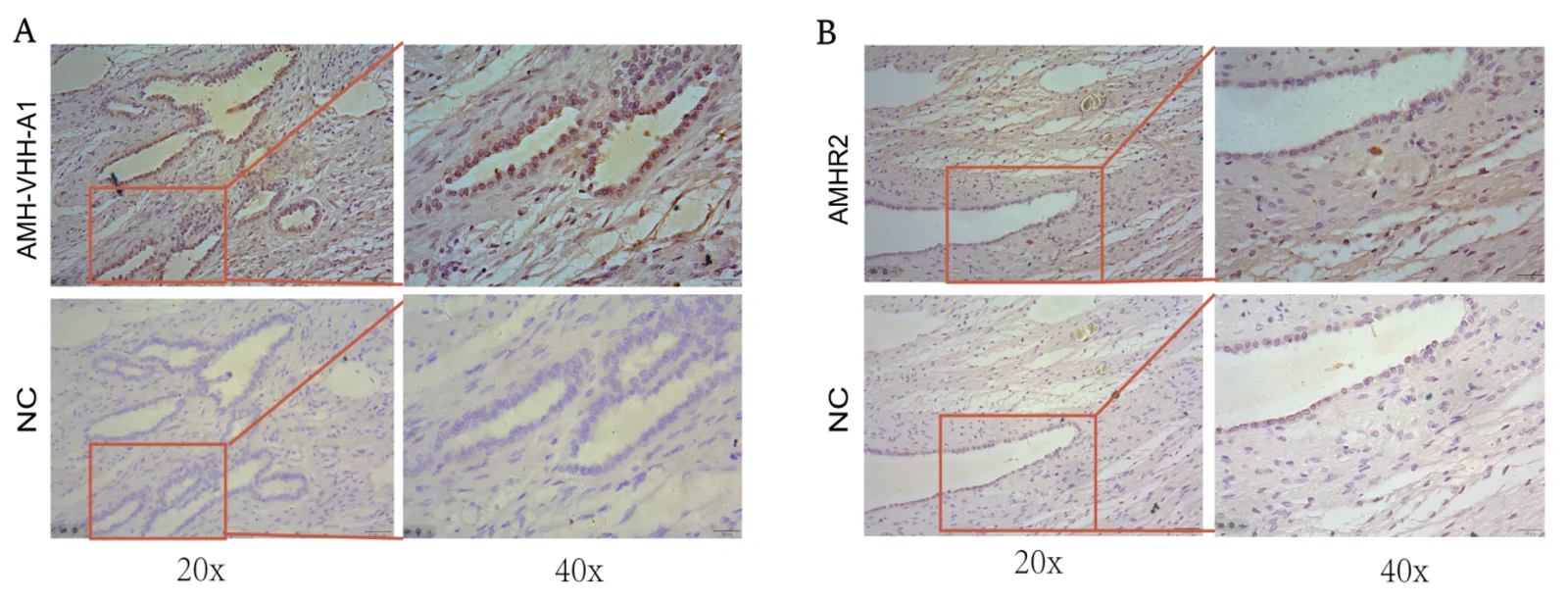
Distribution of AMH and AMHR2 in ovaries of Bactrian camels. (**A**,**B**) Distribution of AMH and AMHR2, respectively, in rete ovarii.

The distribution of AMH (Figure 3A) and AMHR2 (Figure 3B) in the ovaries of Bactrian camels was detected using immunohistochemical methods.

**Figure 4:**
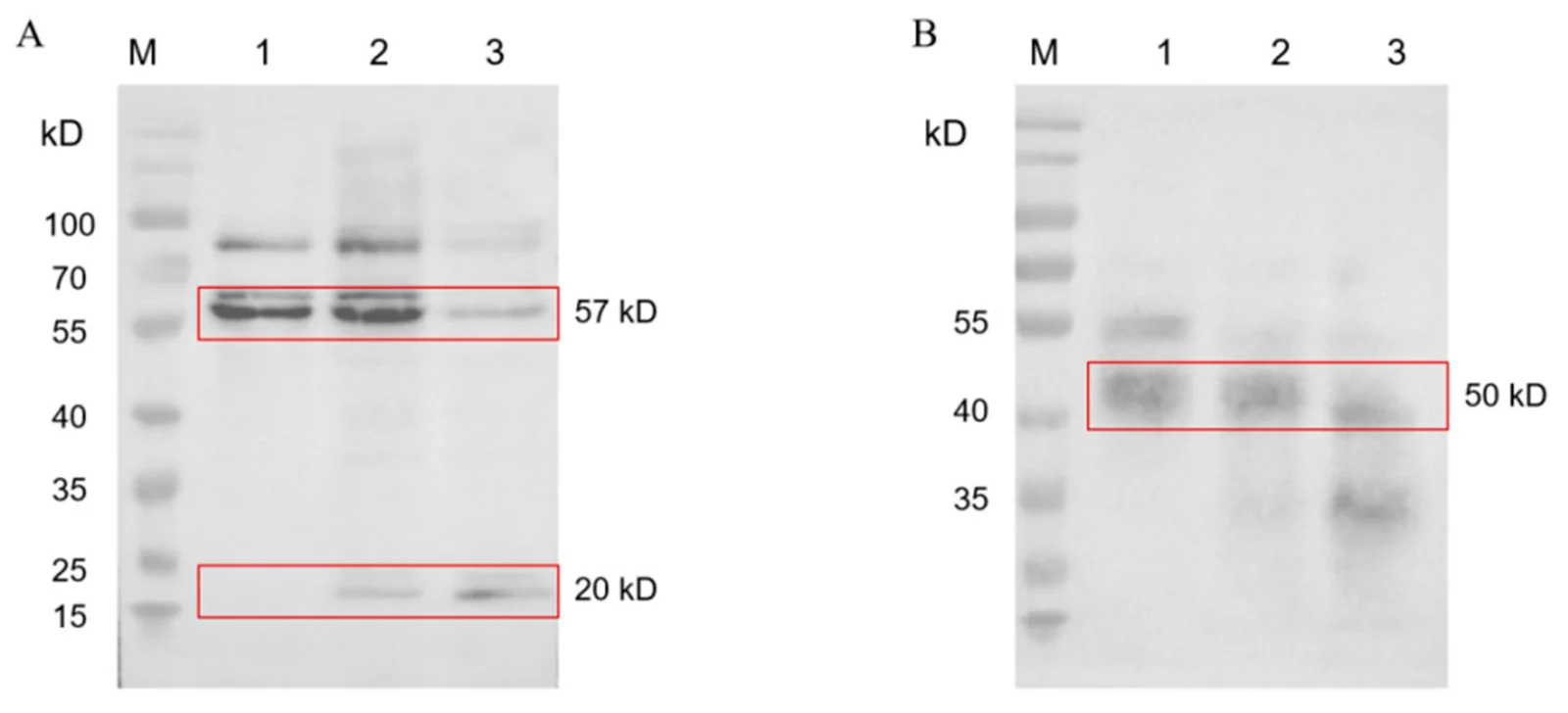
Expression of AMH and AMHR2 in ovaries of Bactrian camels. (**A**,**B**) Expression of AMH and AMHR2, respectively, in ovaries of Bactrian camels (lane M: marker; lanes 1~3: proteins from ovarian tissues of three different camel individuals).

The expression of AMH in the ovaries of Bactrian camels was detected using Western blotting with VHH-AMH-A10, yielding positive bands at approximately 57 kDa and 20 kDa (Figure 4A; Figure S1A). Anti-AMHR2 antibody was used to detect AMHR2, resulting in a positive band at around 50 kDa and AMHR2 (Figure 4B; Figure S1B).

### 3.3. Relatively Quantitative Serum AMH Levels and Amounts of Growing Follicles in Ovaries of Bactrian Camels

**Figure 5:**
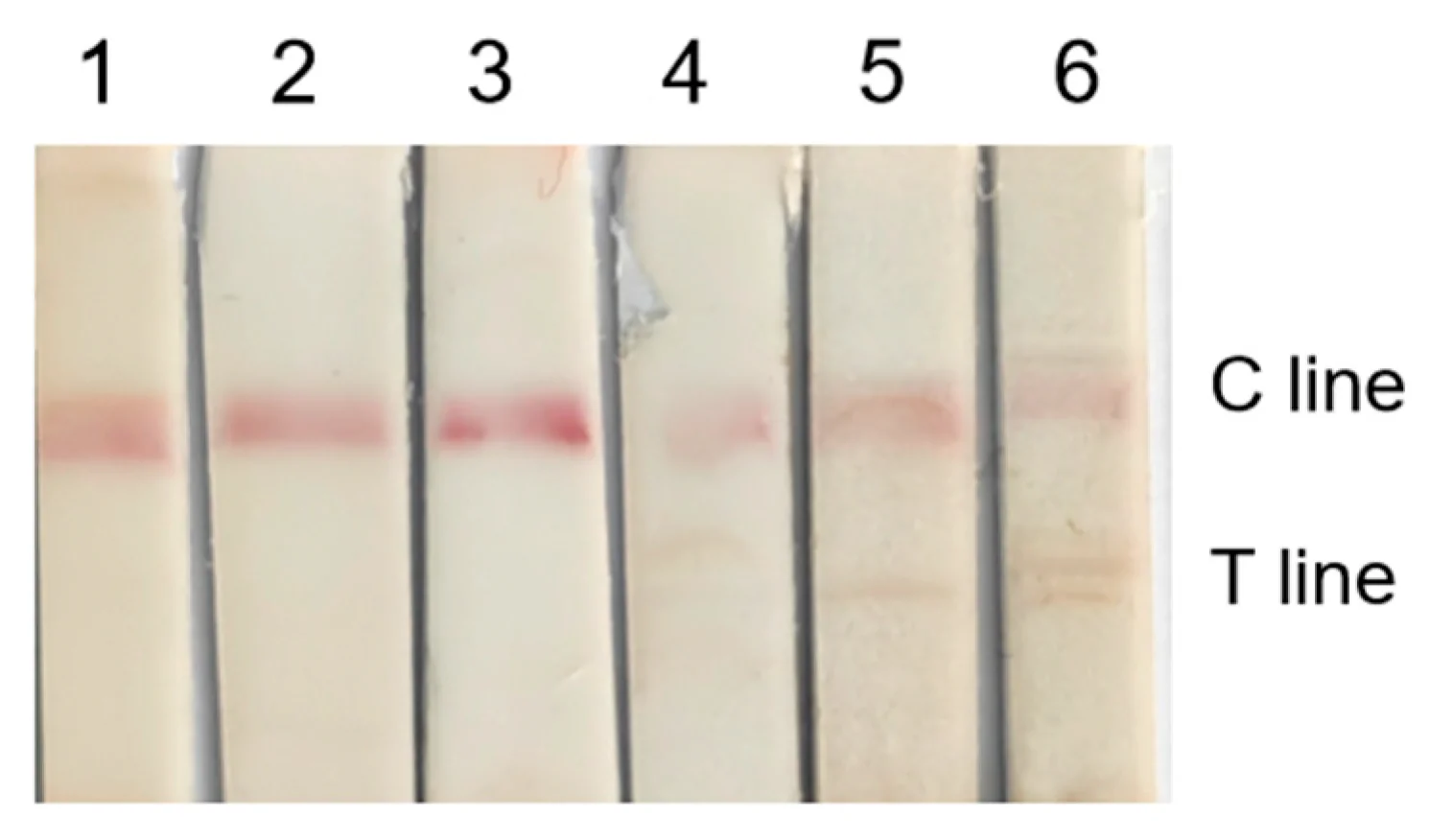
Serum AMH levels in camels detected by colloidal gold immunochromatography, with C line representing the control line, and T line representing the test line.

**Figure 6:**
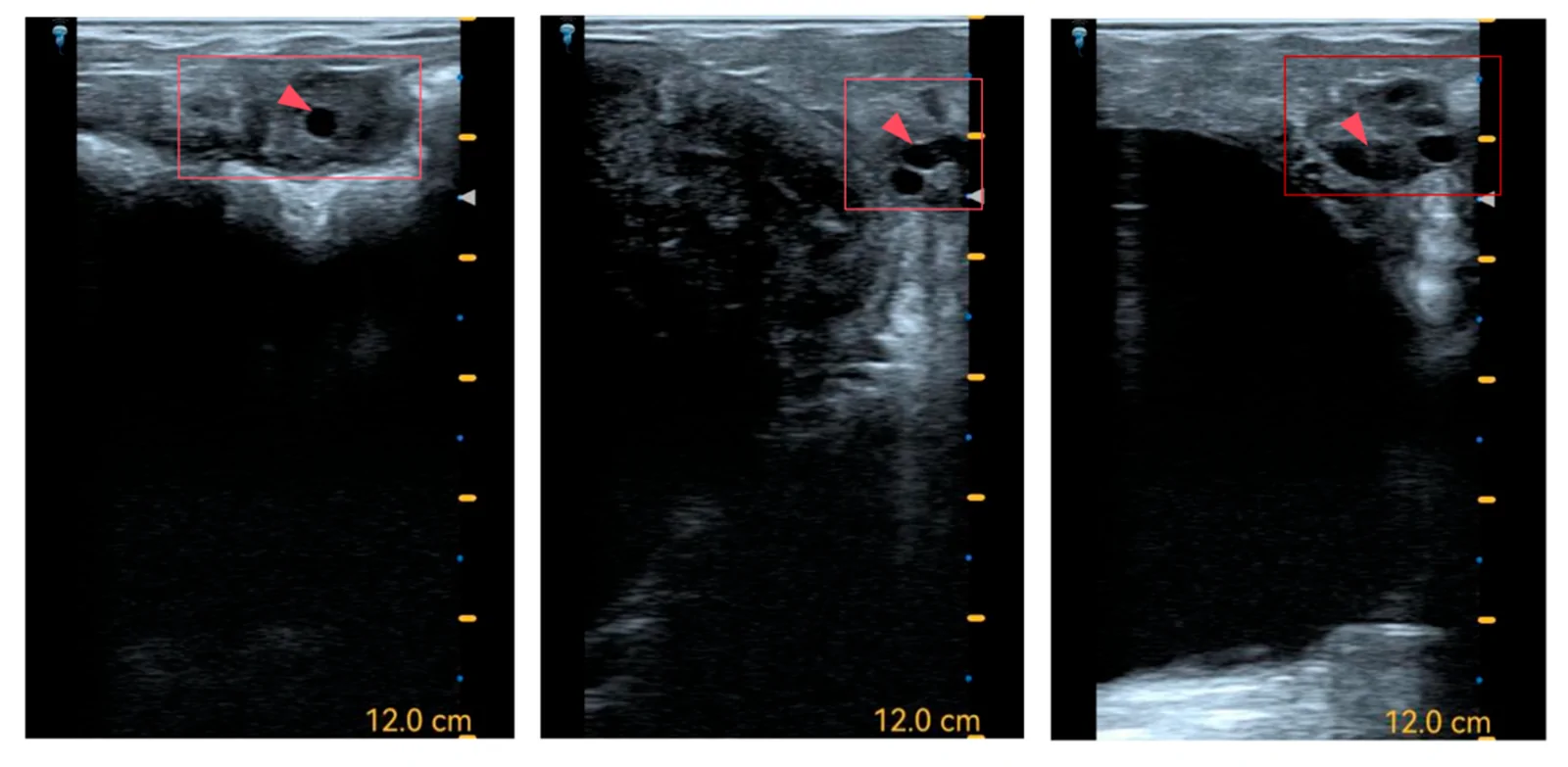
Images of growing follicles in camels detected by Ultrasound B. Growing follicles are shown in the red boxes.

The serum AMH level in camels was measured by the established colloidal gold immunochromatography method based on nanobodies VHH-AMH-A1 and VHH-AMH-A10, and the results showed that the C line and the T line appeared obviously within 3 min (Figure 5). At the same time, images of growing follicles in the corresponding camels were collected by ultrasound B, and the results showed clear bound6aries of growing follicles with a black echo (Figure 6).

## 4. Discussion

In this research, two nanobodies against AMH were obtained to be used in the expression of AMH in Bactrian camel ovaries by the Western blotting method, with positive bands of approximately 57 kDa and 20 kDa. Although camel-derived AMH consists of 570 amino acids, and exhibits 76% amino acid similarity to human AMH, the 20 kDa band observed in the Western blotting results deviated slightly from the theoretical value (12 kDa). Nevertheless, the presence of the 57 kDa band confirmed the expression of the target protein in camel ovarian tissue. Whether the structure with a size of approximately 20 kDa in the ovaries of Bactrian camels is related to the structure with a size of approximately 12 kDa needs further research. Immunohistochemical staining exhibited specific signals, predominantly distributed in the rete ovarii of the ovarian medulla, with AMH and AMHR2 being particularly prominent in the cuboidal epithelial cells, which may be closely associated with their biological functions. Given that the function of the ovarian rete is still unclear, the expression of both AMH and AMHR2 in the epithelial cells of the ovarian rete in Bactrian camels suggests that the ovarian rete might have functional roles in secreting AMH.

The highly consistent AMH value in dromedaries is a reliable indicator for predicting superovulatory responses and results in dromedaries [17]. Based on the specific nanobodies against AMH, this study further established a colloidal gold immunochromatography technique to assess the developmental status of camel follicles. The colloidal gold detection results were consistent with the images of growing camel follicles obtained by B-mode ultrasound imaging, with T-line intensity showing a positive correlation with the number of growing follicles. However, only a limited number of animals were included in the validation phase due to the scarcity of experimental camel resources. Further research is required on AMH levels among camels at different ages and physiological stages.

This immunochromatographic assay reported here does not rely on large professional instruments, and supports rapid on-site detection of serum samples to enable real-time evaluation of follicular development in camels. It meets the demand of Bactrian camel breeders for screening reproductive potential, and it provides intuitive and rapid data support for farm management, including grouping breeding and precise mating, delivering practical value for refined reproductive management in Bactrian camel farming.

## 5. Conclusions

The present research obtained two anti-AMH nanobodies (~15 kDa), which were applied to develop a colloidal gold immunochromatographic strip for evaluating follicular development in Bactrian camels.

## Data Availability

The original contributions presented in this study are included in the article/Supplementary Materials. Further inquiries can be directed to the corresponding authors.

## Supplementary Materials

The following supporting information can be downloaded at: https://www.mdpi.com/article/10.3390/vetsci13080788/s1, Figure S1: The original Western Blot images of the expression of AMH (Fig.A) and AMHR2 (Fig.B) in ovaries of Bactrian camels; Table S1: Immunoreactivity Evaluation of AMH Protein Using ELISA Assay.
