# Supplementary material for: A Method Based on Nanobodies Against AMH to Establish Immunochromatography for Evaluation of Follicular Development in Bactrian Camels
Source: Vet Sci. 2026 Aug 7;13(8):788. doi: 10.3390/vetsci13080788 (PMC13517711; doi:10.3390/vetsci13080788)
Supplement: Supplementary file 1 [file vetsci-13-00788-s001.zip › Table S1.pdf]

Immunoreactivity Evaluation of AMH Protein Using ELISA Assay

<>	1	2	3	4	5	6	7	8	9	10	11	12
A	0.390199989	0.349599987	0.444000006	0.426600009	0.429899991	0.0469	1.100399971	0.564100027	0.311699986	0.422399998	0.474599987	0.729799986
B	0.558499992	0.5079	0.469500005	0.423599988	0.452899992	0.489800006	0.65140003	0.746699989	0.510299981	0.407499999	0.50029999	0.920099974
C	0.447299987	0.420399994	0.450599998	0.552399993	0.321999997	0.053300001	0.957400024	0.432300001	0.494899988	0.925100029	0.709100008	0.115400001
D	0.526300013	0.312900007	0.111500002	0.113799997	0.179700002	0.0528	0.926400006	0.704200029	0.196199998	0.563199997	0.458099991	0.1065
E	0.1118	0.076700002	0.094700001	0.092699997	0.075999998	0.061500002	0.278800011	0.26910001	0.106600001	0.196099997	0.129700005	0.067599997
